# Parametric Representation of Tactile Numerosity in Working Memory

**DOI:** 10.1523/ENEURO.0090-19.2019

**Published:** 2020-02-06

**Authors:** Işıl Uluç, Lisa Alexandria Velenosi, Timo Torsten Schmidt, Felix Blankenburg

**Affiliations:** 1 Neurocomputation and Neuroimaging Unit (NNU), Department of Education and Psychology, Freie Universität Berlin, 14195 Berlin, Germany; 2 Berlin School of Mind and Brain, Humboldt-Universität zu Berlin, 10099 Berlin, Germany

**Keywords:** Working memory, numerosity, tactile, fMRI, MVPA, abstract quantity

## Abstract

Estimated numerosity perception is processed in an approximate number system (ANS) that resembles the perception of a continuous magnitude. The ANS consists of a right lateralized frontoparietal network comprising the lateral prefrontal cortex (LPFC) and the intraparietal sulcus. Although the ANS has been extensively investigated, only a few studies have focused on the mental representation of retained numerosity estimates. Specifically, the underlying mechanisms of estimated numerosity working memory (WM) is unclear. Besides numerosities, as another form of abstract quantity, vibrotactile WM studies provide initial evidence that the right LPFC takes a central role in maintaining magnitudes. In the present fMRI multivariate pattern analysis study, we designed a delayed match-to-numerosity paradigm to test what brain regions retain approximate numerosity memoranda. In line with parametric WM results, our study found numerosity-specific WM representations in the right LPFC as well as in the supplementary motor area and the left premotor cortex extending into the superior frontal gyrus, thus bridging the gap in abstract quantity WM literature.

## Significance Statement

While the perception of approximate numerosities has been extensively investigated, research into the mnemonic representation during working memory (WM) is relatively rare. Here, we present the first study to localize WM information for approximate numerosities using functional magnetic resonance imaging in combination with multivariate pattern analysis (MVPA). Extending beyond previous accounts that used either a priori brain regions or electrocorticography with poor spatial resolution and univariate analysis methods, we used an assumption-free, time-resolved, whole-brain searchlight MVPA approach to identify brain regions that code approximate numerosity WM content. Our findings in line with previous work, provide preliminary evidence for a modality- and format-independent, abstract quantitative WM system, which resides within the right lateral PFC.

## Introduction

Humans can tell whether 100 people are a larger group than 50 people quite accurately without counting. This ability to quantify amount, size, length, or other analog stimulus properties can be performed nonsymbolically, independent of language (Dehaene, 1992; Spitzer et al., 2014b). Indeed, human infants and several animals are able to approximate a variety of quantities (Nieder, 2005; Piazza et al., 2007; Piazza and Izard, 2009; Nieder and Dehaene, 2009), suggesting a common elemental system which has been termed the approximate number system (ANS; Gallistel and Gelman, 1992; Dehaene, 2011).

While numerosity is a discrete stimulus property, the ANS allows an approximation of numerosity, resulting in an analog estimation. Thus, in contrast to the symbolic mental representation of numbers as classes or categories, it has been hypothesized that the ANS representation resembles that of continuous quantities or magnitudes such as intensities, lengths, or frequencies (Piazza et al., 2004; Nieder and Dehaene, 2009; Spitzer et al., 2014a). In support of this, neural representations underlying both the ANS and continuous quantities have been shown to be supramodal, implying a representation abstract in nature (Piazza et al., 2006; Spitzer and Blankenburg, 2012; Spitzer et al., 2014a; Vergara et al., 2016). Moreover, small numbers are rapidly and accurately identified without counting, known as subitizing (Kaufman et al., 1949). Thus, these numbers are represented as discrete values. If the number of items exceeds the subitizing threshold, counting is required to determine the exact amount. When there is insufficient time for counting, the ANS approximates the quantity in a fast and efficient manner.

The functional anatomy of the ANS has been extensively characterized in both human and nonhuman primates (NHPs). A frontoparietal network comprising the dorsolateral prefrontal cortex and the posterior parietal cortex (PPC), specifically the intraparietal sulcus (IPS), is involved in approximating quantities during perception (Dehaene et al., 2004; Piazza et al., 2004, 2007; Cantlon et al., 2006, 2009; Jacob and Nieder, 2009; Knops and Willmes, 2014). Moreover, the right hemisphere has been shown to be dominant with respect to quantity estimation (McGlone and Davidson, 1973; Young and Bion, 1979; Kosslyn et al., 1989); however, recent studies have found that both hemispheres respond to approximate visual numerosity (Piazza et al., 2004; Ansari et al., 2006). Particularly in nonsymbolic numerosity perception, the IPS has been shown to exhibit stronger numerosity-selective responses than the PFC (Tudusciuc and Nieder, 2009), and the PPC, especially the IPS, responds to the nonsymbolic numerosity processing (Piazza et al., 2004, 2007).

The ANS literature is primarily focused on perception with relatively few NHP studies extending to investigate working memory (WM) representations of approximate quantities (Nieder, 2016). As short-term maintenance of information is critical for higher-order cognitive functions such as decision-making and reasoning, it is crucial to investigate beyond perception to the maintenance of approximate quantities in WM. In line with results from perception studies of the ANS, neurons in the frontoparietal network were found, specifically in the PFC and IPS, to exhibit numerosity-selective activity during WM (Jacob et al., 2018). Furthermore, supramodal coding of numerosity memoranda in the frontoparietal cortex has been identified (Nieder, 2017). Interestingly, in contrast to perception, the proportion of numerosity-selective neurons in the PFC and their tuning strength to numerosity have been more prominent than the ones in the PPC during WM retention. Moreover, neurons in the PFC remained selective and discriminated numerosities better than neurons in the PPC during the WM delay (Nieder and Miller, 2004; Tudusciuc and Nieder, 2009; Nieder, 2016).

To the best of our knowledge, only a single study has focused on the WM representation of numerosity in humans (Spitzer et al., 2014a), although some approximate numerosity perception studies used fMRI multivariate pattern analysis (MVPA) method with WM-related paradigms focusing on the perceptual processes instead of the WM retention (Eger et al., 2009; Borghesani et al., 2019; Castaldi et al., 2019). Spitzer et al. (2014a) probed the oscillations underlying multimodal WM representations by training participants to estimate numerosity from sequential auditory, visual, and tactile stimuli. They identified strong and long-lasting alpha oscillations in the PPC reflecting WM load, whereas, in line with NHP results, beta-band activity in the right PFC showed numerosity-selective modulation.

Nevertheless, whole-brain research regarding the localization of numerosity memoranda in humans is lacking. To this end, we designed a tactile delayed match-to-numerosity (DMTN) task in combination with whole-brain, searchlight, MVPA of human fMRI data (Christophel et al., 2012; Schmidt et al., 2017; Uluç et al., 2018). Using this analysis approach, we localized brain regions maintaining approximate number content in WM. As per previous studies (Spitzer et al., 2014a; Nieder, 2016), we hypothesized that the content would be represented in frontal regions, specifically the right PFC.

## Materials and Methods

### Participants

Thirty-eight healthy volunteers participated in the study. The sample size was based on the successful use of similar sample sizes in earlier MVPA experiments with analog experimental designs and analyses (Schmidt et al., 2017; Christophel et al., 2018). In addition, it accords with recent theoretical work on power analysis for random field theory-based cluster-level statistical inference (Ostwald et al., 2019). The data of four participants were excluded due to low performance levels (≤60%), resulting in data from 34 participants (mean ± SD age, 25.53 ± 5.43 years; 19 females) being further analyzed. All were right handed according to the Edinburgh Handedness Inventory with a mean ± SD index of 0.82 ± 0.14 (Oldfield, 1971). The experimental procedure was approved by the local ethics committee and was conducted in accordance with the Human Subject Guidelines of the Declaration of Helsinki. All participants provided written informed consent before the experiment and were compensated for their participation.

### Stimuli

Tactile stimuli consisted of trains of square-wave electric pulses (200 μs) delivered via a pair of surface-adhesive electrodes attached to the participant’s left wrist. A constant current neurostimulator (model DS7A, Digitimer) was used to deliver the stimuli. Subjects reported tactile sensations radiating to the thumb, index, and middle finger, verifying stimulation of the median nerve. Individual sensory thresholds were determined for each participant. The stimulus intensity was then adjusted to a target value of ∼200% of the sensory threshold (mean, 6.42 mA; SD, 1.20 mA).

**Figure 1.**
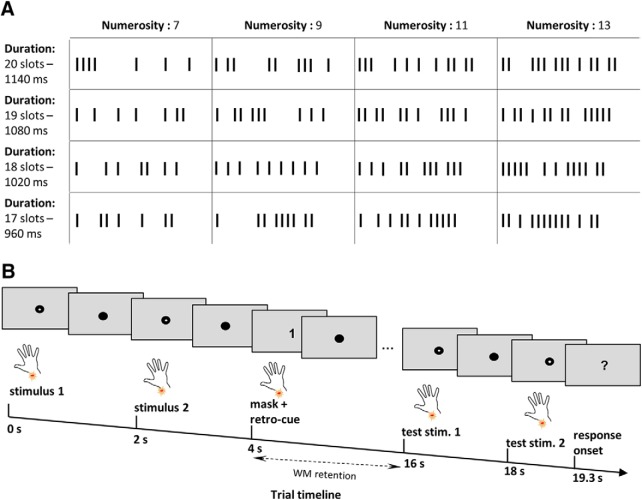
Sample pulse sequences and experimental paradigm. ***A***, Sample stimuli. Pulse sequences of 7, 9, 11, and 13 were used as experimental stimuli. For each numerosity, there were four different durations (960, 1020, 1080, and 1140 ms), where each duration was subdivided into 60 ms slots. The distribution of pulses to slots was randomized for each stimulus presentation. The first and the last slot of each stimulus always contained a pulse. The stimuli displayed are for illustrative purposes. ***B***, Experimental paradigm. A delayed match-to-numerosity task was used, where two sample stimuli and a mask were presented consecutively. A visual retro-cue that was presented simultaneously with the mask indicated which of the numerosities should be retained for the 12 s delay. After the delay, participants performed a two-alternative forced choice, indicating which of the two test stimuli had the same numerosity as the cued stimulus. The response period was 1.5 s. Please note that the stimulus duration and interstimulus interval changed depending on the stimulus duration, but the onset of each event was locked to coincide with the onset of an image acquisition.

A to-be-remembered stimulus sequence comprised 7, 9, 11, or 13 pulses. To dissociate stimulus length and perceived pulse frequency (spacing of tactile pulses) from the numerosity of pulses, the duration of the stimulus varied, and the interpulse intervals were randomized. To this end, we defined four stimulus durations (960, 1020, 1080, and 1140 ms). Each duration was subdivided into 60 ms slots, resulting in 17, 18, 19, and 20 slots, respectively. The temporal distribution of the pulses was then randomized across the slots (Fig. 1*A*, illustrative stimuli). Within each run, each numerosity was presented in a short (17 or 18) and a long (19 or 20) duration, resulting in 24 different numerosity–duration pairings (4 numerosities × 2 durations/run × 3 uncued numerosities). The different durations were balanced across runs. The alternatives for each cued numerosity were computed according to the respective sample (±3 pulses). Additionally, the target stimulus and the cued sample never had the same duration, ensuring that memorizing the duration or average frequency of the target does not help to perform the task. We also performed a Fourier transformation of the stimuli, which ensured that all stimuli were composed of similar combinations of frequencies. Therefore, this stimulus design ensured that participants had to memorize the stimulus numerosity since they could not use the temporal density of the pulses or the stimulus length as WM memoranda to solve the task.

### Task

We used a DMTN paradigm in which participants remembered the estimated numerosity of a stimulus. Each trial began with the presentation of two pulse sequences with different numerosities. Next, a retro-cue (“1” or “2”) indicated which of the two numerosities had to be remembered. To suppress potential perceptual residues, in the sense of afterimages (Sperling, 1960; Christophel and Haynes, 2014; Christophel et al., 2015), a mask consisting of the longest duration (1140 ms) with a pulse in each of the 20 slots, was applied simultaneously with the onset of the retro-cue. Following a 12 s retention phase, two test stimuli were presented and a two-alternative forced choice was given. Neither of the test stimuli were identical to the encoded stimulus; however, one had the same numerosity, while the duration and the frequency were different. This ensured that participants used the approximated numerosity of the stimulus instead of some other stimulus feature to correctly match the test with the remembered stimulus. The numerosity of the alternative stimulus was three pulses plus or minus the target stimulus. To ensure that the number of pulses in a sequence could not be easily counted, the lower alternative stimulus for the lowest to-be-remembered numerosity (7), was set to 5 and thus above a previously established subitizing threshold of approximately 4 (for tactile modality, it was shown to be 3–4; Riggs et al., 2006; Plaisier et al., 2009, 2010; Plaisier and Smeets, 2011; Spitzer et al., 2014a; Tian and Chen, 2018). After the second target stimulus, participants had 1.5 s to indicate, via button press with their right middle or index finger, which of the two stimuli had the same numerosity as the encoded stimulus (Fig. 1*B*, experimental design). Furthermore, the response mapping was counterbalanced across participants. In total, a trial lasted 21 s and an experimental run, consisting of all possible stimulus pairings presented equally often (12 pairings × 4 presentations = 48 trials) in a randomized order, with intertrial intervals of 1.5 or 3.5 s, lasted 18.7 min. Four experimental runs were collected for each participant, resulting in a total recording time of 74.8 min.

Before the fMRI experiment, each participant was familiarized with the timing and structure of the task by performing up to two experimental runs outside the scanner.

### Number naming test assessing countability

Subsequent to the fMRI session, we applied a number-naming task to ensure that participants were unable to count the number of pulses used in the stimulus set. Participants were asked to try to count the number of pulses. The stimuli ranged from 1 to 15 pulses with 5 different duration and temporal pulse distribution combinations of each numerosity were tested, resulting in 75 trials. The counting test was performed after fMRI data acquisition so as to prevent biasing the participants toward counting the pulses in the main experiment.

To ensure that the presented numerosities were above participants’ subitizing thresholds, we calculated the mean performance for each numerosity across participants and calculated each average estimated numerosity. We then compared the slope of accuracy for estimating numerosities with earlier studies that calculated subitizing thresholds for tactile stimuli (Riggs et al., 2006; Plaisier et al., 2009, 2010; Plaisier and Smeets, 2011; Spitzer et al., 2014a; Tian and Chen, 2018). We performed a linear trend analysis using linear regression to determine whether the distance between the true and estimated numerosity scales with increasing true numerosity in a linear fashion.

### fMRI data acquisition and preprocessing

fMRI data were acquired in four runs, with a Siemens 3 T Tim Trio MRI scanner (Siemens) equipped with a 32-channel head coil. In each run, 565 images were collected (T2*-weighted gradient echo EPI: 37 slices; ascending order; 20% gap; whole brain; TR = 2000 ms; TE = 30 ms; 3 × 3 × 3 mm³; flip angle = 70°; 64 × 64 matrix). After the last functional run, a high-resolution structural scan was recorded using a T1-weighted MPRAGE sequence (1 × 1 × 1 mm³; TR = 1900 ms; TE = 2.52 ms; 176 sagittal slices). fMRI data preprocessing was performed using SPM12 (Wellcome Trust Center for Neuroimaging, Institute for Neurology, University College London, London, UK). Functional images were slice time corrected and spatially realigned to the mean image. To conserve the spatiotemporal structure of the fMRI data for the multivariate analyses, no smoothing or normalization was performed. For the univariate control analysis, functional images were normalized to MNI space and smoothed with an 8 mm FWHM kernel.

### First-level finite impulse response models

A time-resolved, multivariate searchlight analysis (Kriegeskorte et al., 2006; Schmidt et al., 2017) was used to identify brain regions encoding memorized numerosity information. First, a general linear model (GLM) with a set of finite impulse response (FIR) regressors was fit to each participant’s data to obtain runwise parameter estimates of each WM content (numerosity value of 7, 9, 11, or 13). A single FIR regressor was estimated for each fMRI image or 2 s time bin (1 TR); thus, the 20 s trial was divided into 10 time bins. We additionally included the first five principal components accounting for the most variance in the CSF and white matter signal time courses, respectively (Behzadi et al., 2007), and six head motion regressors, as regressors of no interest. Moreover, the data were filtered with a high-pass filter of 128 s. The resulting parameter estimates were used for the MVPA, performed with The Decoding Toolbox (TDT) version 3.52 (Hebart et al., 2015).

### Multivariate pattern analysis

For the decoding of memorized numerosity information, a searchlight-based multivariate analysis using a support vector regression (SVR) approach was performed with the computational routines of LIBSVM (Chang and Lin, 2011), as implemented in TDT. SVR MVPA (for more discussion, see Kahnt et al., 2011; Schmidt et al., 2017) considers the variable of interest (memorized numerosity) as a continuous data vector with multiple independent variables (multivariate BOLD activities) as opposed to the commonly used support vector machine approach that treats the variable of interest as a categorical object. This means that the SVR MVPA approach seeks a linear continuum for the numerosities in which their distance is proportional to the distances of the rank order.

We analyzed each time bin independently by implementing a searchlight decoding analysis with a spherical searchlight radius of 4 voxels. For a given voxel, *z*-scaled parameter estimates (across samples) corresponding to each WM condition were extracted from all voxels within the spherical searchlight for each run. This yielded 16 pattern vectors (4 WM contents × 4 runs), each corresponding to the BOLD activity pattern for a specific WM condition of a functional run. We then fitted a linear function to these pattern vectors such that the multivariate distribution for the different numerosities follows a linear mapping of numerosities. The *z*-scaled parameter estimates were entered into an SVR model with a fixed regularization parameter c that was set to 1.

We used a leave-one-run-out cross-validation scheme for the subject-level decoding analysis. The SVR classifier was trained on three runs (12 pattern vectors) and tested on the data of the independent fourth run (4 pattern vectors) for how well it predicted the values of the remaining run. The allocation of training and test runs was iterated so that each of the four functional runs was used as a test run once, resulting in four cross-validation folds. The prediction performance from each cross-validation fold was reported by a Fisher’s *z*-transformed correlation coefficient between the predicted and the actual numerosity information estimate. The mean prediction accuracy across cross-validation folds was assigned to the center voxel of the searchlight, and the center of the searchlight was moved voxel by voxel through the brain, resulting in a whole-brain prediction accuracy map. Consequently, we obtained one prediction accuracy map for each time bin for each participant, where the prediction accuracy reflects how well a linear ordering according to the associated numerosities could be read out from the locally distributed BOLD activity pattern at a given voxel location and time.

Next, prediction accuracy maps were normalized to MNI space and smoothed with an 8 mm FWHM kernel. They were then entered into a second-level, repeated-measures ANOVA with subject and time (time bins) as factors. To assess which brain regions exhibit WM content-specific activation patterns during the delay period, we computed a *t*-contrast across the six time bins corresponding to the 12 s WM delay (time bins 3–8). The results are presented at *p* < 0.05 family-wise error (FWE) correction at the cluster level with a cluster-defining threshold of *p* < 0.001. Cytoarchitectonic references are based on the SPM anatomy toolbox where possible (Eickhoff et al., 2005). Presented images (e.g., surface projections with applied color scales) were created using MRIcron version9/9/2016 (McCausland Center for Brain Imaging, University of South Carolina, Columbia, SC).

### Control analyses

In the first control analysis, we examined whether the decoded numerosity information during WM retention was specific to WM or could be assigned to perceptual residues. To this aim, we defined a second, first-level model with FIR regressors for the nonmemorized stimulus. We then implemented the identical searchlight decoding procedure as the main analysis. Thus, this control analysis tested for the presence of numerosity information of the nonmemorized stimulus.

Next, we conducted a parametric univariate analysis to ensure that the decoded information in the main analysis is not due to the modulation of mean activity level. To this end, we fitted a standard GLM with the following four HRF-convolved regressors: one regressor to capture WM processes, a parametrically modulated regressor for the numerosity content of the WM memoranda as well as eight [4 numerosities × 2 (sample, test)] additional parametrically modulated regressors for each sample and test stimulus. First-level baseline contrasts for the parametric effect of memorized numerosity were forwarded to a second-level one-sample *t* test.

Finally, to test the specificity of the SVR analysis to the parametric order of the four numerosities, we performed exhaustive whole-brain SVR searchlight analyses for all possible permutations of numerosity labels. To achieve this, we computed distance rank order as a sum of the absolute difference of adjacent ranks [e.g., 11, 13, 7, and 9 numerosity is distance 5 (|3–4|+|4–1|+|1–2|)] for all possible permutations of the numerosity order. Then, the permutations were grouped according to their distance from the original rank order. We used 12 instead of 24 permutations as the distances of rank order permutations are symmetric. Including the permutation with the correct linear order, the 12 permutations are aggregated into five classes depending on their distance from the correct linear order. Then, for each permutation analysis, we extracted the prediction accuracies of the group-peak voxels that are defined in the original analysis. For statistical assessment, we calculated the mean prediction accuracy across related time bins (WM time bins 3–8) for each peak voxel for each distance group (see Fig. 3*C*).

## Results

### Behavioral performance

**Figure 2.**
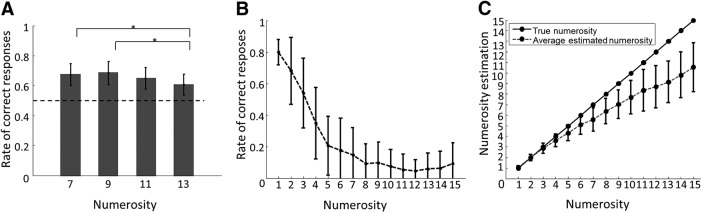
***A***, Mean rate of correct responses across participants (*n* = 34) for different numerosities in the main WM DMTN task. The figure shows that the WM performance decreases with increasing numerosity. Error bars represent standard derivation (SD). Asterisks indicate statistical significance for pairwise *t* tests, Bonferroni corrected for multiple comparisons (*p* < 0.05/6). ***B***, Mean performance across subjects for estimated numerosity in number naming task (mean ± SD). ***C***, True numerosities versus mean numerosity estimations (error bars show SD).

Thirty-four participants performed with 65.36 ± 3.29% (mean ± SD) accuracy in the demanding DMTN task across the four experimental runs (Fig. 2*A*). To test whether the behavioral performance differed for the four numerosity values, we performed a one-way repeated-measures ANOVA with four levels, one for each numerosity. This test revealed a significant main effect (*F*_(3,135)_ = 7.52, *p* < 0.001). *Post hoc t* tests (Bonferroni corrected for multiple comparisons) between performances were significant for numerosity values 7 and 13 and 9 and 13 (*p* < 0.05/6; Fig. 2*A*). This is expected because we did not control for the Weber–Fechner effect except for the lowest numerosity (which we did due to subitizing concerns). As a result, as the numerosity increases, it becomes more difficult to differentiate between the sample and alternative stimuli, thus resulting in a lower performance for high numerosities (Fechner, 1966) but is unlikely to affect WM processing.

### Behavioral performance on number naming test assessing countability

To test whether participants were able to count the numerosities used in the current study, participants performed an additional number-naming test. Previous research in tactile numerosity indicated the subitizing threshold for comparable stimuli to be four pulses (Riggs et al., 2006; Plaisier et al., 2009; 2010; Plaisier and Smeets, 2011; Spitzer et al., 2014a; Tian and Chen, 2018). The approximation of the subitizing threshold identified in the present study is in line with these reports (Fig. 2*B*). As expected, participants’ perceptual accuracy decreased with increasing numerosity, and performance decreased to 50% when more than three pulses were presented. Similarly, the distance between the true and estimated numerosity increased with increasing numerosities (*p* < 0.001, linear trend analysis; Fig. 2*C*).

### Multivariate mapping of regions that code numerosity as WM content

**Figure 3.**
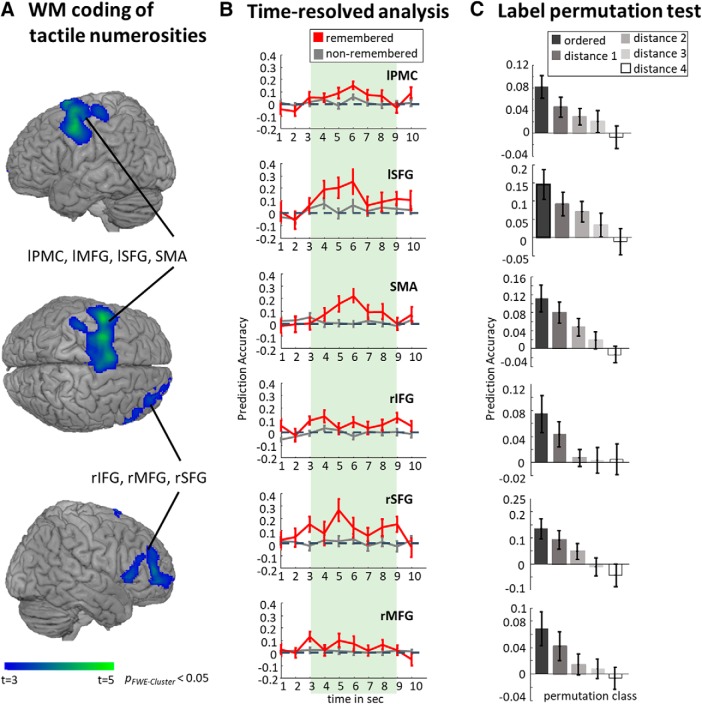
***A***, Brain regions coding information for the memorized estimated numerosities. Group-level results of a *t*-contrast testing the 12 s WM delay for above-chance prediction accuracy. Brain regions carrying information about memorized scalar magnitudes are as follows: IFG, MFG, PMC, SMA, and SFG. ***B***, Time courses of decoding accuracies of remembered (red) and nonremembered (gray) stimuli for all identified brain regions in the main analysis (Fig. 3*A*). Error bars indicate standard error of the mean (SEM). The figure shows that, for all clusters depicted in the main analysis, there is more numerosity-specific WM information for the remembered than for the forgotten numerosity, and the information is present throughout the WM delay period. ***C***, Results of the label permutation tests. Five bars are shown for each brain region, respectively. Each bar displays the mean prediction accuracy estimated from the distance to correct order groups. The shade of the bar color, ranging from black to white, depicts the different distance to correct ordering. Black bars indicate the mean prediction performance of the group with the correct linear order, while white bars represent the mean prediction accuracy derived from the most linearly unordered data. Brain regions tested for label permutation are: IFG, MFG, PMC, SMA, and SFG. Error bars indicate SEM.

The time-resolved, searchlight-based multivariate regression analysis was performed to identify brain regions representing estimated numerosity memoranda. The SVR MVPA analysis for the WM retention period revealed numerosity-specific responses in the left premotor cortex (PMC) slightly extending to the primary motor area (MI), left middle frontal gyrus (MFG), left superior frontal gyrus (SFG) extending into bilateral supplementary motor areas (SMA), right SFG extending to the right frontal pole, and right MFG extending into the pars triangularis of the right inferior frontal gyrus (IFG). Results are reported at *p* < 0.05, FWE corrected at the cluster level with a cluster-defining threshold of *p* < 0.001 (Fig. 3, Table 1).

**Table 1:** Anatomic label and MNI coordinates of brain areas depicting memorized numerosity information during WM

		**Peak MNI coordinates**				
**Cluster size**	**Anatomical region**	***x***	***y***	***z***	***z*-score**	**Prediction accuracy**
4557	Left PMC/MI	−50	2	52	4.78	0.082
	Left SFG	−28	0	60	7.74	0.146
	SMA	−6	10	74	4.48	0.114
1342	Right SFG	32	50	10	4.17	0.135
	Right IFG (pars triangularis)	60	24	2	4.17	0.075
	Right MFG	40	50	30	3.69	0.069

All results are reported at *p*_FWE-Cluster_ < 0.05 with a cluster-defining threshold of *p* < 0.001. Mean prediction accuracy over the delay period is reported. Areas were, where possible, identified using the SPM anatomy toolbox (Eickhoff et al., 2005).

For the sake of completeness, we investigated whether numerosity information could be decoded from the IPS at an uncorrected statistical threshold of *p* < 0.001. We found a cluster in the right PPC extending to the IPS (peak at MNI: *x* = 36, *y* = −52, *z* = 36 mm; *z* score = 3.89; *k* = 164), which was identified as hIP1 with a 39.5% probability and hIP3 with a 5.9% probability using the SPM anatomy toolbox (Eickhoff et al., 2005) at *p*_uncorrected_ < 0.001.

### Control analyses

To test whether the identified decoded information is indeed specific to the memorized numerosity representation, we applied the same searchlight procedure to the nonmemorized numerosity stimulus. This analysis did not reveal any clusters with above-chance prediction accuracy at p_FWE-Cluster_ < 0.05.

Additionally, we conducted a univariate parametric analysis to test whether the decoding results could be due to differences in activation strength between WM contents. A second level *t* test revealed no significant voxels at *p*_FWE-Cluster_ < 0.05, thus providing evidence for the multivariate nature of the numerosity representations identified in this study rather than the modulation of univariate mean activity.

Finally, we performed label-permutation tests to ensure the specificity of the linear ordering of stimuli in the SVR MVPA. Higher prediction accuracies were expected when the activation patterns in a given brain region represented the correct order of the four numerosity labels, and it was expected to decrease with the distance from the correct ordering. As expected, the prediction accuracy during WM was the highest for the true-labeled data and decreased with increasing distance from the correct ordering (Fig. 3*C*).

## Discussion

The current study, to our knowledge, is the first to identify brain regions that code approximate numerosity WM content using human neuroimaging methods. Thus, this study extends the broad literature on ANS perception to the maintenance of mental representations, which can be used for higher-order cognitive functions. We used a well established, whole-brain, searchlight, DMTN paradigm to identify representations of tactile approximate numerosity memoranda. Specifically, we used an SVR technique, which, in contrast to support vector machines, treats the retained WM content as a continuous variable and thus predicts the ordering of content along the variable, rather than a singularly specific class label. Consequently, an above-chance prediction accuracy in a brain region means that the content-specific activation patterns follow a linear ordering according to the associated numerosity. Our searchlight analysis identified a distributed network spanning the left PMC, bilateral SFG, bilateral SMA, and right MFG extending into right IFG. Therefore, these regions contain linearly ordered, multivariate WM representations of the numerosities.

Our results are in line with previous numerosity WM studies in NHPs and human EEG, which have established the central role of the PFC. Indeed, previous unimodal and multimodal studies have identified content-specific representations in the PFC (Nieder and Miller, 2004; Tudusciuc and Nieder, 2009; Spitzer et al., 2014a; Nieder, 2016; Jacob et al., 2018). More specifically, in humans, parametric modulation of upper-β oscillations in the right lateral PFC has been shown to reflect analog numerosity estimation that has been derived from discrete sequences, both within and between stimulus modalities (Spitzer et al., 2014a). Thus, the numerosity representations in the PFC are likely to be supramodal in nature. However, those studies used either electrophysiological recordings from an a priori brain region or have used univariate data analysis methods. The present study extends the literature on numerosity WM in the following two ways: first, to whole-brain fMRI data; and second, to multivariate data analysis methods, specifically the SVR MVPA. The benefits of multivariate over univariate analysis methods have been well established (Haynes, 2015). Multivariate analysis techniques are sensitive to the combinatorial aspects of voxel activity, thereby enabling the identification of spatially distributed representations (Haynes, 2015; Hebart and Baker, 2018). Thus, our results agree with and extend the previous NHP and human EEG numerosity WM findings to whole-brain, spatially distributed activity patterns, suggesting that estimated numerosity WM content is maintained in the LPFC (Nieder et al., 2002; Nieder and Miller, 2003, 2004; Tudusciuc and Nieder, 2009; Spitzer et al., 2014a; Nieder, 2016).

It should be noted that we used temporally distributed tactile numerosity stimuli as the WM memoranda, namely the numerosity, was presented as a sequence of pulses. Evidence exists for potential differences in perceptual processing of spatially and temporally distributed numerosities, where spatially distributed stimuli appear to be processed in parietal regions while temporarily distributed stimuli do not (Cavdaroglu and Knops, 2019). In line with the finding of Cavdaroglu and Knops (2019), we used temporally distributed stimuli and did not find evidence of WM representations in posterior regions in our full brain FWE-corrected analysis. However, a small cluster (*k* = 164) extending to right IPS was observed to represent remembered numerosity content at an uncorrected threshold of *p* < 0.001. While our results agree with numerosity WM findings in NHPs that suggest frontal rather than parietal coding for spatial numerosity stimuli during WM retention (for review, see Nieder, 2016), further investigation is needed to conclusively decide for the role of the IPS. The role of the IPS could be interpreted as specific to perceptual processing, and therefore was only revealed at a lower threshold in our analysis, while the PFC contains WM instead. Alternatively, a potentially different nature of the neuronal code (e.g. spatial distribution of a multivariate code) in the IPS might lead to the observed findings (Hebart and Baker, 2018). That is, it might be the temporarily distributed nature of the applied stimuli that drives the effects in the PFC, and the IPS would be more specialized for spatially distributed presentations as used by most previous studies. A future direct comparison of our results with spatial numerosity stimuli is necessary to test for differences determined by the stimulus types.

Moreover, while the literature relating to numerosity WM is limited, there is extensive work exploring the WM representation of abstract quantities more generally. Specifically, the frequency discrimination task has been systematically explored in a multitude of modalities with a wide range of methods (Romo et al., 1999; Lemus et al., 2009; Spitzer et al., 2010; Spitzer and Blankenburg, 2011; 2012; Fassihi et al., 2014; Vergara et al., 2016; Schmidt et al., 2017; von Lautz et al., 2017; Uluç et al., 2018; Wu et al., 2018). Numerosity and frequency share several traits, particularly that they are both abstract magnitudes that may be represented in a supramodal fashion (Nieder and Miller, 2003; Spitzer and Blankenburg, 2012; Nieder, 2016; Vergara et al., 2016). However, whether their underlying WM representations are maintained by a shared network has yet to be explored. The present study provides an initial step toward resolving this question by providing the first evidence that frequency and numerosity WM representations are maintained in overlapping brain regions. We identified numerosity-specific WM content in the right IFG, SMA, and left PMC, which is in agreement with results from frequency studies also using an fMRI MVPA approach in humans (Schmidt et al., 2017; Wu et al., 2018; Uluç et al., 2018). Unimodal and multimodal research in both NHPs and humans has identified frequency-specific content in the right LPFC and SMA, thereby suggesting that the WM representations are modality independent in nature (Romo et al., 1999; Hernández et al., 2002, 2010; Barak et al., 2010; Spitzer et al., 2010; Spitzer and Blankenburg, 2011, 2012; Vergara et al., 2016; Schmidt et al., 2017; Wu et al., 2018). However, the explicit relationship between frequency and numerosity still needs to be explored, particularly with respect to the underlying neural codes of numerosity and frequency representations (Nieder, 2017).

Additionally, we identified numerosity-specific content in the left PMC. Previous findings from frequency WM fMRI MVPA studies identified abstract quantity information in the PMC (Schmidt et al., 2017; Uluç et al., 2018; Wu et al., 2018) . Moreover, the dorsal PMC has been shown to represent abstract numerical rules, such as comparison and calculation (Gruber et al., 2001; Eger et al., 2003; Nieder, 2005). This is in line with the present task, which required the comparison of numerical quantities, suggesting representation of task-relevant, numerosity-specific information to be used in numerical comparison.

In summary, the data at hand is in line with the suggestion of a domain general, abstract magnitude processing system. This abstract processing system can be identified by multivariate WM representations of tactile numerosity stimuli within the right PFC. Together with previous findings that found WM representations of tactile frequency (Spitzer et al., 2010, 2014a; Spitzer and Blankenburg, 2012; Schmidt et al., 2017; Wu et al., 2018), visual flicker frequency (Spitzer and Blankenburg, 2012; Spitzer et al., 2014a; Wu et al., 2018), auditory frequency (Spitzer and Blankenburg, 2012, Uluç et al., 2018), and the reports of number coding (Nieder et al., 2002; Nieder and Miller, 2003, 2004; Tudusciuc and Nieder, 2009; Nieder, 2016) in the PFC, the present study provides additional evidence suggesting that the PFC is capable of representing both analog quantities as well as parametric stimulus properties as frequencies. Thus, we provide preliminary evidence for a higher-level, modality- and format-independent, abstract quantitative WM system that resides within the PFC.

## Synthesis

Reviewing Editor: Bradley Postle, University of Wisconsin Decisions are customarily a result of the Reviewing Editor and the peer reviewers coming together and discussing their recommendations until a consensus is reached. When revisions are invited, a fact-based synthesis statement explaining their decision and outlining what is needed to prepare a revision will be listed below. The following reviewer(s) agreed to reveal their identity: André Knops.

Here is the full text of the two reviews:

Reviewer #1

Summary

In this study, the authors investigate the neural correlates of numerosity information that is maintained in working memory. Participants were sequentially presented with two trains of tactile numerosities (7, 9, 11, or 13), followed by a cue indicating which one participants should remember. After a masking train and a delay period of 12s, participants decided which of two probes would correspond to the maintained numerosity. Accuracy decreased with increasing numerosity. Using SVR, authors identified a set of six frontal regions that exhibited linear relationship between numerosity and activity patterns. Based on these findings, authors argue for a pivotal role of frontal brain structures (PMA, SMA, rIFG, rMFG) in the maintenance of tactile numerosity information in working memory. In particular, authors argue that these findings imply “a higher level, modality- and format independent abstract quantitative WM system which resides within the right lateral PFC.”

Evaluation

The study addresses an interesting and timely topic and fills an important empirical gap by focusing on the interaction between numerosity perception and working memory mechanisms. The study design is well-thought, the stimulus sets attempts to control for the most important confounds between numerosity and other non-numerical features of the trains. Analysis mainly focuses on MVPA using SVR which poses higher constraints on the data structure and hence is a more conservative but more stringent approach compared to SVC. The general claims are supported by the results but require corroboration from additional analyses. Theoretically, the repeated references to subitizing do neither fit within the context of the current paper nor do the data actually allow to draw this parallel between visual and tactile enumeration.

Major comments

42: Subitizing is a phenomenon that is different from the numerosity estimation that invokes the ANS. Subitizing hinges on object individuation system and is functionally linked to saliency maps. Subitizing is attentionally demanding while estimation is not (estimation was not impacted by a attention grabbing dual-task while subitizing limit decreased under dual-task condition).

68f: “Interestingly, in contrast to perception, the proportion of numerosity selective neurons and their tuning strength to numerosity have been more prominent than the ones in the PPC during WM retention” Please check. Do authors refer to PFC neurons here?

89: How was sample size determined? What was the particular reason for choosing 34 participants?

121: “To ensure the dissociation between perceptual processes and the memory-related activity, a mask consisting of the longest duration (1140 ms) with a pulse in each of the 20 slots, was applied simultaneously with the onset of the retro-cue”. I am not sure I understand the rationale here. How does presenting a constant stimulus that “masks” the sensory input help to dissociate perceptual from memory-related activity? If it is meant to ‘erase’ the activity related to perception then this would not be in line with the concept of additivity of BOLD response in fMRI. That is, the BOLD response to S1 would simply be S1+mask, S2 would be S2+mask. So given a constant interval between masks and S1 and S2, I do not understand the rationale here. Please clarify.

129: I always wondered whether the subitizing phenomenon would actually have a correspondence in the tactile domain. Unfortunately, none of the references provided here actually establishes this. Lechelt presented stimuli in the range 3 < S <10 only. Schmidt et al. presented stimuli in the frequency range 10Hz - 46 Hz, not analyzing individual numerosity of the trains. Only Philippi et al. (2008) show a linear relationship between presented and observed numerosity when ISI equaled 320ms. Yet, below this ISI numerosity estimates were underestimated, contradicting the robust subitizing phenomenon in the visual domain. This coincides with the data you report in Figure 2A. Participants start at values well below 100% accuracy for even 1 stimulus. Immediately accuracy decreases as numerosity increases. This is different from subitizing where accuracy and RTs are largely unaffected (and close to 100%) by numerical magnitude in this range. Overall, the decrease of accuracy and increase of RT appear to follow exponential patterns rather than showing two different regimes as is the case in subitizing. Together, I think it is rather misleading to speak about subitizing in the tactile domain.

195: The description of data input to the MVPA classifier is not clear to me. With the designdescribed in line 195, authors achieve highly volatile parameter estimates per condition that are subsequently fed into the MVPA classifier. Yet, later (210f) they report to rather rely on more stable parameter estimates and fewer partitions of the data (i.e. 4 runs). Please clarify (deleting the sentence in line 195 would suffice).

Additional analysis: Given that authors went to great pains using FIR modeling to capture the time-course of BOLD activity I found it disappointing to not see those data depicted in the manuscript. I think the manuscript would benefit from including the following two figures/analyses. 1) For each of the six ROIs a figure depicting the time-course for the four to-be-remembered numerosities. In particular, working memory literature suggests sustained activity as one of the key mechanisms for maintenance of information in working memory. Hence, we would stipulate to see sustained activity during maintenance period. 2) For each of the six ROIs a time-resolved MVPA analysis depicting how decoding accuracy evolves over the course of time (i.e. when does it reach its maximum)? Sustained activity should lead to high decoding accuracy over the course of time. In line with the idea that information decays over time, we would predict a general decrease of decodability of numerosity information towards the end of the retention period. Together, I think these analyses would be an asset to the manuscript and provide additional empirical underpinning to the idea that these ROIs code tactile numerosity during working memory maintenance.

Furthermore, it is necessary to show that the regions identified by the classifier are activated in the first place. While I agree that the multivariate analysis does not stipulate a parametric modulation of activity with numerosity, the regions should at least be activated in general (i.e. in an F-contrast, for example or testing all numerosities against zero).

Reviewer #2

The paper reports one fMRI experiment where adult human subjects evaluate the relative numerosity of two sequences of tactile stimuli. Specifically, the experiment was designed in order to evaluate, using MVPA, the pattern of BOLD activity corresponding to the period where the first stimulus is maintained in short term memory, before the second stimulus appears, thus probing the neural representation of tactile numerosity in memory.

The methods and analyses appear sound, and the results convincing.

However, I have a request for additional information on the stimuli: while it is clear at at the level of decision making subjects could not rely on the frequency or on the duration of the stimuli (as these features varied across sample and match trials), it is not clear whether the frequencies characterizing the different numerosities to be retained in memory, systematically varied (on average) with number or not. Looking at the figure and reading the methods it may seem that, on average, larger numerosities were also characterized by higher frequencies. Was this the case? If that was the case, there might be a problem for interpreting the results, unless the authors concentrate on a specific sub-set of controlled stimuli. However, if number is not overall correlated with frequency then the current interpretation of the results is valid, and I suggest that the authors provide with a table with average frequency, and average duration for the different numerosities so that the stimulus space is transparent to the reader.

A second point that I would like to comment relates to the literature review, which I did not always found appropriate:

1) On the right lateralization of the ANS : it is claimed that the ANS consists of a right lateralized frontoparietal network comprising the lateral prefrontal cortex (LPFC) and the intraparietal sulcus. I do not see how, given the current empirical data, we can be so sure on the right lateralization of the ANS. In order to support this claim the authors refer to Piazza et al., 2006 and Dehaene, 2016. The cited Dehaene 2016 was not present in the references and therefore I was not able to evaluate it. The Piazza 2006 was a classical subtraction-based fMRI which is known not to be very sensitive, thus subject to false negative results. Thus, the fact that Piazza et al. found higher activity during estimation in the right vs. left IPS cannot, to my understanding, be taken as a demonstration that the ANS is right lateralized. On the contrary, there is a great deal of research using fMRI that do show bilateral acrivation as supporting numerosity representations. In sum, I would be more cautious in making strong claims relative to lateralization for the ANS.

2) On the fact that the IPS exhibit stronger numerosity-selective responses than the PFC.

To support this claim the authors refer to Dehaene et al., 2003; Eger et al., 2009.

However, those citations are not appropriate: Dehaene et al., 2003 is a meta-analysis and they did not show any data comparing PFC vs. IPS in terms of numerosity-selective response. Eger et al., 2009 only scanned parietal cortex, so they could not compare numerosity decoding based on parietal vs. pre-frontal activation (from the Eger paper: “Of note, we did not cover homologous frontal regions in our scanning volume”).

3) On the fact that there is no previous whole brain MVPA study of working memory maintenance of numerosity information: they authors could refer to a recently published work that uses a delayed number comparison task that requires holding numerosity information in WM (Borghesani, V. et al. Processing number and length in the parietal cortex: Sharing resources, not a common code. Cortex (2018)).

Finally, I would like to make a point on the results, and in particular about the absence of a significant effect of decodability of numerosity based on parietal cortex activation. Because the absence of numerosity-dependent signal in parietal cortex is a negative finding, and the statistical thresholds used to report the whole brain results rather strict, I wonder if

a lower statistical threshold would reveal a hint of numerosity coding in parietal cortex as well or not.

I think that the field would profit from this supplementary analysis in order to have a better understanding of whether parietal cortex involvement in numerosity estimation is solely confined to simultaneously presented items (as also previously suggested by Knops), and to what extent this representation of fully absent when subjects are dealing with sequentially presented items.

## Author Response

We thank the reviewers for their thorough and helpful comments. Here are our responses to their inquiries:

Reviewer #1

Major comments

42: Subitizing is a phenomenon that is different from the numerosity estimation that invokes the ANS. Subitizing

hinges on object individuation system and is functionally linked to saliency maps. Subitizing is attentionally demanding while estimation is not (estimation was not impacted by an attention grabbing dual-task while subitizing limit decreased under dual-task condition).

We agree with the reviewer that subitizing and estimation are two different processes. For this reason, we changed the corresponding text to read:

Moreover, small numbers are rapidly and accurately identified without counting, known as subitizing (Kaufman et al., 1949).

68f: “Interestingly, in contrast to perception, the proportion of numerosity selective neurons and their tuning strength to numerosity have been more prominent than the ones in the PPC during WM retention” Please check. Do authors refer to PFC neurons here?

We would like to thank the reviewer for pointing out the missing words in the sentence that created the confusion. We changed it to:

Interestingly, in contrast to perception, the proportion of numerosity selective neurons in the PFC and their tuning strength to numerosity have been more prominent than the ones in the PPC during WM retention.

89: How was sample size determined? What was the particular reason for choosing 34 participants?

We based our sample size on previous studies using similar experimental designs and analyses. In addition, our study complies with recent sample size calculations indicating that

30-50 participants are needed for a desirable maximal power of 0.8 at medium effect sizes for random field theory-based cluster-level inference (Ostwald et al., 2019). We revised the manuscript in order to clarify the motivation of the sample size:

Participants

38 healthy volunteers participated in the current study. The sample size was based on the successful use of similar sample sizes in earlier MVPA experiments with analog experimental designs and analyses (e.g., Schmidt et al., 2017; Christophel et al., 2018). In addition, it accords with recent theoretical work on power analysis for random field theory-based cluster-level statistical inference (Ostwald et al., 2019). The data of four participants had to be excluded due to low performance levels ({less than or equal to} 60%) resulting in a sample size of 34 participants (age: 25.53 {plus minus} 5.43 mean years {plus minus} SD, 19 females).

Christophel, T. B., Allefeld, C., Endisch, C., & Haynes, J.-D. (2018). View-Independent Working Memory Representations of Artificial Shapes in Prefrontal and Posterior Regions of the Human Brain. Cerebral Cortex, 28(6), 2146-2161. https://doi.org/10.1093/cercor/bhx119

Ostwald, D., Schneider, S., Bruckner, R., & Horvath, L. (2019). Power, positive predictive value, and sample size calculations for random field theory-based fMRI inference. bioRxiv, 613331. https://doi.org/10.1101/613331

121: “To ensure the dissociation between perceptual processes and the memory-related activity, a mask consisting of the longest duration (1140 ms) with a pulse in each of the 20 slots, was applied simultaneously with the onset of the retro-cue”. I am not sure I understand the rationale here. How does presenting a constant stimulus that “masks” the sensory input help to dissociate perceptual from memory-related activity? If it is meant to ‘erase’ the activity related to perception then this would not be in line with the concept of additivity of BOLD response in fMRI. That is, the BOLD response to S1 would simply be S1+mask, S2 would be S2+mask. So given a constant interval between masks and S1 and S2, I do not understand the rationale here. Please clarify.

We thank the reviewer for drawing our attention to this potentially confusing formulation. The purpose of the mask is not to influence the BOLD signal resulting from perception but to remove potential perceptual residues, in the sense of afterimages. Using a mask to suppress such perceptual residues has been successfully used in in working memory experiments in the visual (Sperling, 1960; Christophel et al., 2012; 2014; 2015) and tactile modality (Schmidt et al., 2017, 2018).

We have now improved the wording to better explain the rationale behind the mask:

Next, a retro-cue (“1” or “2”) indicated which of the two numerosities had to be remembered. To suppress potential perceptual residues, in the sense of afterimages (e.g. Sperling, 1960; Christophel and Haynes, 2014; Christophel et al., 2015) , a mask consisting of the longest duration (1140 ms) with a pulse in each of the 20 slots, was applied simultaneously with the onset of the retro-cue.

References

Christophel, T.B., Haynes, J.-D. (2014). Decoding complex flow-field patterns in visual working memory. Neuroimage 91, 43-51. doi:10.1016/j.neuroimage.2014.01.025

Christophel, T.B., Cichy, R.M., Hebart, M.N., Haynes, J.-D. (2015). Parietal and early visual cortices encode working memory content across mental transformations. Neuroimage 106, 198-206. doi:10.1016/j.neuroimage.2014.11.018

Sperling, G. (1960). The information available in brief visual presentations.

Psychological Monographs: General and Applied, 74(11), 1-29.

129: I always wondered whether the subitizing phenomenon would actually have a correspondence in the tactile domain. Unfortunately, none of the references provided here actually establishes this. Lechelt presented stimuli in the range 3 < S <10 only. Schmidt et al. presented stimuli in the frequency range 10Hz - 46 Hz, not

analyzing individual numerosity of the trains. Only Philippi et al. (2008) show a linear relationship between presented and observed numerosity when ISI equaled 320ms. Yet, below this ISI numerosity estimates were underestimated, contradicting the robust subitizing phenomenon in the visual domain. This coincides with the data you report in Figure 2A. Participants start at values well below 100% accuracy for even 1 stimulus. Immediately accuracy decreases as numerosity increases. This is different from subitizing where accuracy and RTs are largely unaffected (and close to 100%) by numerical magnitude in this range. Overall, the decrease of accuracy and increase of RT appear to follow exponential patterns rather than showing two different regimes as is the case in subitizing. Together, I think it is rather misleading to speak about subitizing in the tactile domain.

Due to the reviewer's comment, we conducted a more thorough literature review on tactile subitizing and identified additional relevant references. Several studies have shown that there is indeed subitizing in the tactile domain (Riggs et al., 2006; Plaisier et al., 2009; 2010;

2011; Tian & Chen, 2018). Of relevance to the present study, Spitzer and colleagues (2014a)

investigated subitizing in the tactile modality using a paradigm very similar to the one implemented in the present study. Moreover, a recent study found that dual task paradigms affect tactile subitizing but not tactile estimation (Tian & Chen, 2018). However, we would also like to point out that the goal of our control experiment was not to measure the subitizing threshold, but to ensure that participants could not count the number of pulses in a sequence. We thank the reviewer for pointing out the lack of more appropriate references and potentially misleading wording. We changed the references regarding tactile subitizing throughout the manuscript. The manuscript now reads:

The numerosity of the alternative stimulus was 3 pulses {plus minus} the target stimulus. To ensure that the number of pulses in a sequence could not be easily counted,the lower alternative stimulus for the lowest to-be-remembered numerosity (7), was set to five(5)and thus above a previously established subitizing threshold of around four (for tactile modality, it was shown to be 3-4; e.g., Riggs et al., 2006; Plaisier et al., 2009;

2010; 2011; Spitzer et al., 2014a; Tian&Chen, 2018). After the second target stimulus, participants had 1.5 s to indicate, via button-press with their right middle or index finger, which of the two stimuli had the same numerosity as the encoded stimulus(see Figure 1B for experimental design).

Number naming test assessing countability

We then compared the slope of accuracy for estimating numerosities with earlier studies that calculated subitizing thresholds for tactile stimuli (Riggs et al., 2006; Plaisier et al., 2009; 2010; 2011; Spitzer et al., 2014a; Tian & Chen, 2018).

Behavioral performance on number naming test assessing countability

To test whether participants were able to count the numerosities employed in the current study, participants performed an additionally number naming test. Previous research in tactile numerosity indicated the subitizing threshold for comparable stimuli to be 4 pulses (Riggs et al., 2006; Plaisier et al., 2009; 2010; 2011; Spitzer et al., 2014a; Tian & Chen, 2018).

Plaisier, M.A., Bergmann Tiest, W. M., & Kappers, A. M. L. (2009). One, two, three, many - Subitizing in active touch. Acta Psychologica, 131. 163-170. doi:10.1016/j.actpsy.2009.04.003.

Plaisier, M.A., Bergmann Tiest, W. M., & Kappers, A. M. L. (2010). Range dependent processing of visual numerosity: similarities across vision and haptics. Experimental Brain Research, 204. 525-537. doi:10.1007/s00221-010-2319-y. PMC 2903696.

Plaisier, M.A., & Smeets, J. B. J. (2011). Haptic subitizing across the fingers. Attention, Perception, & Psychophysics, 73. 1579-1585. doi:10.3758/s13414-011-0124-8. PMC

3118010.

Riggs, K.J., Ferrand, L., Lancelin, D., Fryziel, L., Dumur, G., & Simpson, A. (2006). Subitizing in tactile perception. Psychological Science, 17(4). 271-272. doi:10.1111/j.1467-9280.2006.01696.x. PMID 16623680.

Tian, Y. & Chen, L. (2018). Cross-modal attention modulates tactile subitizing but not tactile numerosity estimation. Attention, Perception, & Psychophysics, 80. 1229. https://doi.org/10.3758/s13414-018-1507-x

195: The description of data input to the MVPA classifier is not clear to me. With the design described in line

195, authors achieve highly volatile parameter estimates per condition that are subsequently fed into the MVPA classifier. Yet, later (210f) they report to rather rely on more stable parameter estimates and fewer partitions of the data (i.e. 4 runs). Please clarify (deleting the sentence in line 195 would suffice).

We thank the reviewer for pointing out the potential confusion. We decoded four numerosities (classes) using a cross-validated support vector regression analysis with finite impulse response (FIR) regressors for each image separately (time bin). 10 FIR regressors were used to model the working memory delay and 2 time bins before and after. For each participant, four independent runs of experimental data were collected. Thus, in each cross- validation step, the support vector regression analysis was trained using 12 data points (4 classes x 3 runs) and tested on its ability to generalize to the fourth run, the 4 remaining data points. This resulted in a whole-brain, participant-specific, multivariate numerosity WM information image for each time bin.

The FIR regressors are run-wise beta estimates based on 12 trial repetitions per run (for a more detailed discussion on the reliability of beta estimates see; Hebart et al., 2014). Moreover, for each time bin, a separate beta estimate is estimated. In lines 196-197, the total number of beta estimates (for all time bins, all numerosities and all runs and all nuisance regressors) were given. In line 212, we tried to clarify that the analysis was done separately for each time bin. Therefore, in line 215 (earlier line 210), we stated that 16 pattern vectors were used (4 WM content regressors x 4 runs) for each time bin. We deleted the sentence in line 195 to avoid future confusion. The manuscript now reads:

We additionally included the first five principal components accounting for the most variance in the cerebrospinal fluid (CSF) and white matter signal time courses respectively (Behzadi et al., 2007), and six head motion regressors, as regressors of no

interest. Moreover, the data was filtered with a high-pass filter of 128 s. The resulting parameter estimates were used for the MVPA performed with The Decoding Toolbox v.

3.52 (TDT) (Hebart et al., 2015).

References

Hebart, M. N., Schriever, Y., Donner, T. H., & Haynes, J.-D. (2014). The Relationship between Perceptual Decision Variables and Confidence in the Human Brain. Cerebral Cortex, 26(1), 118-130. doi:10.1093/cercor/bhu181

Additional analysis: Given that authors went to great pains using FIR modeling to capture the time-course of BOLD activity I found it disappointing to not see those data depicted in the manuscript. I think the manuscript would benefit from including the following two figures/analyses. 1) For each of the six ROIs a figure depicting the time-course for the four to-be-remembered numerosities. In particular, working memory literature suggests sustained activity as one of the key mechanisms for maintenance of information in working memory. Hence, we would stipulate to see sustained activity during maintenance period. 2) For each of the six ROIs a time-resolved MVPA analysis depicting how decoding accuracy evolves over the course of time (i.e. when does it reach its maximum)? Sustained activity should lead to high decoding accuracy over the course of time. In line with the idea that information decays over time, we would predict a general decrease of decodability of numerosity information towards the end of the retention period. Together, I think these analyses would be an asset to the manuscript and provide additional empirical underpinning to the idea that these ROIs code tactile numerosity during working memory maintenance. Furthermore, it is necessary to show that the regions identified by the classifier are activated in the first place. While I agree that the multivariate analysis does not stipulate a parametric modulation of activity with numerosity, the regions should at least be activated in general (i.e. in an F-contrast, for example or testing all numerosities against zero).

We thank the reviewer for their useful suggestion. It should be noted that while MVPA results can be sensitive to multidimensional processes, univariate voxel-wise analysis cannot since the univariate analysis results show overall mean activation whereas multivariate analysis reflects the spatially-distributed activity patterns. Additionally, with relatively short ITI's and a high temporal correlation between events, the paradigm was optimized for an MVPA analysis rather than a univariate analysis. Hence, the regions identified by the MVPA will not necessarily be identified by a univariate analysis. Therefore, we did not report the univariate analysis results. However, the reviewer is correct by stating that the prediction accuracies for the remembered stimuli should indeed be above chance during the maintenance period. Additionally, to ensure the decoding reflects the memory trace, the prediction accuracy for the non-remembered stimuli should be at chance level. Thus, we modified the manuscript to include the time courses of the prediction accuracies for the remembered and non-remembered stimuli for the 6 ROIs. The figure is updated as follows:

Fig. 3. A. Brain regions coding information for the memorized estimated numerosities. Group level results of a t-contrast testing the 12 s WM delay for above chance prediction accuracy. Brain regions carrying information about memorized scalar magnitudes are: IFG = inferior frontal gyrus, MFG = middle frontal gyrus, PMC = premotor cortex, SMA = supplementary motor area, SFG = superior frontal gyrus. B. Time- courses of decoding accuracies of remembered (red) and non-remembered (grey) stimuli for all identified brain regions in the main analysis (Fig. 3B). Error bars indicate standard error. The figure shows that, for all clusters depicted in the main analysis, there is more numerosity-specific WM information for the remembered than forgotten numerosity and the information is present throughout the WM delay period. C. Results of the label-permutation tests. 5 bars are shown for each brain region, respectively. Each bar displays

the mean prediction accuracy estimated from the distance to correct order groups. The shade of the bar color, ranging from black to white, depicts the different distance to correct ordering. Black bars indicate the mean prediction performance of the group with the correct linear order, while white bars represent the mean prediction accuracy derived from the most linearly unordered data. Brain regions tested for label permutation are: IFG = inferior frontal gyrus, MFG = middle frontal gyrus, PMC = premotor cortex, SMA = supplementary motor area, SFG = superior frontal gyrus. Error bars indicate standard error of the mean.

Reviewer #2

The paper reports one fMRI experiment where adult human subjects evaluate the relative numerosity of two sequences of tactile stimuli. Specifically, the experiment was designed in order to evaluate, using MVPA, the pattern of BOLD activity corresponding to the period where the first stimulus is maintained in short term memory, before the second stimulus appears, thus probing the neural representation of tactile numerosity in memory.

The methods and analyses appear sound, and the results convincing.

However, I have a request for additional information on the stimuli: while it is clear at at the level of decision making subjects could not rely on the frequency or on the duration of the stimuli (as these features varied across sample and match trials), it is not clear whether the frequencies characterizing the different numerosities to be retained in memory, systematically varied (on average) with number or not. Looking at the figure and reading the methods it may seem that, on average, larger numerosities were also characterized by higher frequencies. Was this the case? If that was the case, there might be a problem for interpreting the results, unless the authors concentrate on a specific sub-set of controlled stimuli. However, if number is not overall correlated with frequency then the current interpretation of the results is valid, and I suggest that the authors provide with a table with average frequency, and average duration for the different numerosities so that the stimulus space is transparent to the reader.

We thank the reviewer for this detailed question. In order to properly address this comment, we performed a Fourier transform on all participant-specific stimuli for each numerosity separately. The numerosities are characterized by, on average, the same frequencies. The averaged power spectra for the stimuli with different numerosities show that the main peak at 16.6 Hz corresponds to the inverse of the inter-pulse interval (1/ 60 ms). Thus, it is very unlikely that the participants used frequency as a proxy for numerosity.

However, it might be even more important to note that the remembered numerosity has not been compared to other remembered samples. Each remembered sample has alternative stimuli (± 3 pulses) and sample stimuli are compared to their respective alternatives (lines

128-129). Additionally, the target stimulus and the remembered sample stimulus never share the same duration (thus, same average frequency/pulse density - lines 125-126). On the other hand, the alternative stimulus could be more similar to the remembered sample in terms of having the same average frequency/pulse density (e.g., a sample stimulus with numerosity 13 and duration 17 slots can have alternative numerosity 16 with duration 20 slots, and a test stimulus numerosity 13 and duration 19 slots). Therefore, the frequency of a stimulus cannot be used to remember the sample or detect the test stimulus. To make the point more clear, we further clarified the stimuli in Methods section. The manuscript reads:

Each duration was subdivided into 60 ms slots, resulting in 17, 18, 19 and 20 slots, respectively. The temporal distribution of the pulses was then randomized across the slots (see Figure 1A for illustrative stimuli). Within each run, each numerosity was presented in a short (17 or 18) and a long (19 or 20) duration resulting in 24 different numerosity-duration pairings (4 numerosities x 2 durations/run x 3 uncued numerosities). The different durations were balanced across runs. The alternatives for each cued numerosity were computed according to the respective sample ({plus minus} 3 pulses). Additionally, the target stimulus and the cued sample never had the same duration ensuring that memorizing the duration or average frequency of the target does not help to perform the task. We also performed a Fourier transformation of the stimuli, which ensured that all stimuli were composed of similar combinations of frequencies. Therefore, this stimulus design ensured that participants had to memorize the stimulus numerosity since they could not use the temporal density of the pulses or the stimulus length as WM memoranda to solve the task.

A second point that I would like to comment relates to the literature review, which I did not always found appropriate:

1) On the right lateralization of the ANS : it is claimed that the ANS consists of a right lateralized frontoparietal network comprising the lateral prefrontal cortex (LPFC) and the intraparietal sulcus. I do not see how, given the current empirical data, we can be so sure on the right lateralization of the ANS. In order to support this claim the authors refer to Piazza et al., 2006 and Dehaene, 2016. The cited Dehaene 2016 was not present in the references and therefore I was not able to evaluate it. The Piazza 2006 was a classical subtraction-based fMRI which is known not to be very sensitive, thus subject to false negative results. Thus, the fact that Piazza et al. found higher activity during estimation in the right vs. left IPS cannot, to my understanding, be taken as a demonstration that the ANS is right lateralized. On the contrary, there is a great deal of research using fMRI that do show bilateral acrivation as supporting numerosity representations. In sum, I would be more cautious in making strong claims relative to lateralization for the ANS.

We are very grateful to the reviewer for making us aware of the unclear references for the right lateralization of the ANS. The right lateralization of estimation has indeed been conclusively shown in several additional studies (Kosslyn et al., 1989; McGlone and Davidson,

1973; Young and Bion, 1979). As explained in detail in the study of Piazza and Collagues

(2006):

“Initially, neuropsychological studies showed that impairments in numerosity estimation are more likely to occur after right than left hemisphere damage (Kimura, 1996; McFie et al., 1950). Later it was shown that the right parietal lobe was the only locus relevant for estimation performance (Warrington and James, 1967), since, out of a pool of subjects with lesions in the three lobes of the two hemispheres, only the group with lesions in the right parietal were impaired at numerosity estimation. Indeed, a right hemisphere superiority in quantity estimation was replicated using unilateral tachis- toscopic presentation of stimuli to normal subjects (Kosslyn et al., 1989a; McGlone and Davidson, 1973; Young and Bion, 1979).” (Piazza et al., 2006)

On the other hand, the reviewer's point towards research showing bilateral activation as supporting numerosity representations is also important. To make the point clearer, we have reformulated also our introduction to now read as follows (lines 54-56):

Moreover, the right hemisphere has been shown to be dominant with respect to quantity estimation (Kosslyn et al., 1989; McGlone and Davidson, 1973; Young and Bion, 1979), however recent studies have found that both hemispheres respond to approximate visual numerosity (Ansari et al., 2006; Piazza et al., 2004).

References

Ansari, D., Dhital, B., Siong, S.C. (2006). Parametric effects of numerical distance on the intraparietal sulcus during passive viewing of rapid numerosity changes. Brain Res.

1067, 181-188.

Kosslyn, S.M., Koenig, O., Barrett, A., Cave, C.B., Tang, J., Gabrieli, J.D. (1989). Evidence for two types of spatial representations: hemispheric specialization for categorical and coordinate relations. J. Exp. Psychol. Hum. Percept. Perform. 15, 723-735.

McGlone, J., Davidson, W. (1973). The relation between cerebral speech laterality and spatial ability with special reference to sex and hand preference. Neuropsychologia 11,

105-113.

Young, A.W., Bion, P.J. (1979). Hemispheric laterality effects in the enumeration of visually presented collections of dots by children. Neuropsychologia 17, 99-102.

2) On the fact that the IPS exhibit stronger numerosity-selective responses than the PFC. To support this claim the authors refer to Dehaene et al., 2003; Eger et al., 2009. However, those citations are not appropriate: Dehaene et al., 2003 is a meta-analysis and they did not show any data comparing PFC vs. IPS in terms of numerosity-selective response. Eger et al., 2009 only scanned parietal cortex, so they could not compare numerosity decoding based on parietal vs. pre-frontal activation (from the Eger paper: “Of note, we did not cover homologous frontal regions in our scanning volume”).

The reviewer is right in their criticism that the cited Eger and colleagues (2009) study, although they performed an exploratory searchlight analysis and found parietal cortex to primarily carry numerosity information, they did not include PFC in their scanning/analysis:

“To further clarify the topographical distribution of numerosity information, we conducted a multivariate “searchlight” analysis [26], testing for the local presence of number information in a sphere with 3 voxel radius sequentially moved across all voxels (27 slices covering parietal and superior parts of the frontal lobes). The resulting maps of classification accuracy scores for each voxel and subject were submitted to a group analysis for both same and different sample stimulus lists. Number information was detected most significantly in parts of the intraparietal sulcus, although at a lower, uncorrected level of significance, additional foci appeared in medial parietal cortex and medial and lateral premotor regions (Figure 3; Table 1). This demonstrates that information discriminating individual numbers is not distributed nonspecifically across wide regions of cortex. Instead, within the limits of our imaging volume, numerical information is mainly concentrated in the intraparietal sulcus, where monkey electrophysiology has identified a high proportion of numerosity-sensitive neurons [8].”

On the other hand, in a neurophysiological study, Tudusciuc and Nieder (2009) showed that during perception, IPS neurons discriminated quantities better than PFC neurons:

“Both length and numerosity were coded by tuning functions peaking at the preferred quantity, thus supporting a labeled-line code for continuous and discrete quantity. A comparison of the response characteristics between parietal and frontal areas revealed a larger proportion of IPS neurons representing each quantity type in the early sample phase, in addition to shorter response latencies to quantity for IPS neurons. Moreover, IPS neurons discriminated quantities during the sample phase better than PFC neurons, as quantified by the receiver operating characteristic area.”

Additionally, Piazza and colleagues (2004; 2007) showed in whole brain univariate analyses that mainly the PPC is activated during non-symbolic numerosity perception. These studies showed that the parietal cortex, especially IPS, is responsive to the non- symbolic numerosity. In line with the suggestion of the reviewer, we changed the citations and the manuscript now reads:

Particularly in non-symbolic numerosity perception, the IPS has been shown to exhibit stronger numerosity-selective responses than the PFC (Tudusciuc and Nieder, 2009) and the PPC, and especially IPS, responds to the non-symbolic numerosity processing (Piazza et al., 2004; Piazza et al., 2007).

3) On the fact that there is no previous whole brain MVPA study of working memory maintenance of numerosity information: they authors could refer to a recently published work that uses a delayed number comparison task that requires holding numerosity information in WM (Borghesani, V. et al. Processing number and length in the parietal cortex: Sharing resources, not a common code. Cortex (2018)).

We greatly thank the reviewer for pointing us to this article. The above-mentioned study, as Eger and colleagues did in 2009, focused their analysis not only on active maintenance but mainly on the stimulus presentation period. We added the study accordingly. The manuscript now reads:

To the best of our knowledge, only a single study has focused on the WM representation of numerosity in humans, although some approximate numerosity perception and numerosity-length comparison studies used fMRI-MVPA method with WM-related paradigms focusing on the perceptual processes instead of the WM retention (e.g., Eger et al., 2009; Borghesani, V. et al., 2018; Castaldi et al., 2019).

References

Borghesani, V., Dolores de Hevia, M., Viarouge, A., Chagas, P. P., Eger, E., & Piazza, M. (2018). Processing number and length in the parietal cortex: Sharing resources, not a common code. Cortex. doi:10.1016/j.cortex.2018.07.017

Castaldi, E., Piazza, M., Dehaene, S., Vignaud, A., & Eger, E. (2019). Attentional amplification of neural codes for number independent of other quantities along the dorsal visual stream. bioRxiv, 527119. https://doi.org/10.1101/527119

Finally, I would like to make a point on the results, and in particular about the absence of a significant effect of decodability of numerosity based on parietal cortex activation. Because the absence of numerosity-dependent signal in parietal cortex is a negative finding, and the statistical thresholds used to report the whole brain results rather strict, I wonder if a lower statistical threshold would reveal a hint of numerosity coding in parietal cortex as well or not. I think that the field would profit from this supplementary analysis in order to have a better understanding of whether parietal cortex involvement in numerosity estimation is solely confined to simultaneously presented items (as also previously suggested by Knops), and to what extent this representation of fully absent when subjects are dealing with sequentially presented items.

We would like to thank the reviewer for further inquiry. We agree that it is difficult to interpret negative results. It is particularly difficult to interpret them in MVPA since the sensitivity of fMRI-MVPA is dependent on the spatial characteristics of the neuronal patterns that represent the feature-specific information. Thus, following the reviewer's suggestion, we also used a lower statistical threshold to investigate whether the PPC carries numerosity WM information. Indeed, there was a small cluster in the right PPC at the uncorrected statistical threshold.

A cluster extending to the right IPS is found at t-score = 3.94, k=164. We added the information to the manuscript. However, it should be noted that the cluster is not very reliable, not overlapping with earlier studies finding IPS in numerosity perception. Thus, not to overemphasize the finding, we briefly mention it in the manuscript. The manuscript now reads:

Results

The time-resolved, searchlight-based multivariate regression analysis was performed to

identify brain regions representing estimated numerosity memoranda. The SVR MVPA analysis for the WM retention period revealed numerosity-specific responses in the left PMC, left middle frontal gyrus (MFG), left superior frontal gyrus (SFG) extending into bilateral supplementary motor areas (SMA), right SFG extending to the right frontal pole and right MFG extending into the pars triangularis of the right IFG. Results are reported at p < 0.05, FWE-corrected at the cluster level with a cluster-defining threshold of p < 0.001 (Figure 3 and Table 1).

For the sake of completeness, we investigated whether numerosity information could be decoded from the IPS at an uncorrected statistical threshold of p < 0.001. We found a cluster in the right PPC extending to the IPS (peak at MNI x = 36, y = -52, z = 36 mm, z- score = 3.89, k = 164), which was identified as hIP1 with a 39.5% probability and hIP3 with a 5.9% probability using the SPM anatomy toolbox (Eickhoff et al., 2005) at puncorrected < 0.001.

[...]

Discussion

Evidence exists for potential differences in perceptual processing of spatially- and temporally-distributed numerosities, where spatially-distributed stimuli appear to be

processed in parietal regions while temporarily-distributed stimuli do not (Cavdaroglu and Knops, 2018). In line with the finding of Cavdaroglu and Knops (2018), we used temporally distributed stimuli and did not find evidence of WM representations in posterior regions in our full brain FWE corrected analysis. However, a small cluster (k=164) extending to right IPS was observed to represent remembered numerosity content at an uncorrected threshold of p < 0.001. While our results agree with numerosity WM findings in NHPs that suggest frontal rather than parietal coding for spatial numerosity stimuli during WM retention (for review, see Nieder, 2016) further investigation is needed to conclusively decide for the role of the IPS. The role of the IPS could be interpreted as specific to perceptual processing, and therefore only revealed at a lower threshold in our analysis, while the PFC contains WM instead. Alternatively, a potentially different nature of the neuronal code, e.g. spatial distribution of a multivariate code, in the IPS might lead to the observed findings (see Hebart and Baker,

2018). That is, it might be the temporarily-distributed nature of the applied stimuli that

drives the effects in the PFC, and the IPS would be more specialized for spatially distributed presentations as used by most previous studies. A future direct comparison of our results with spatial numerosity stimuli is necessary to test for differences determined by the stimulus types.

We thank the reviewers for these thorough and very useful suggestions once more. Sincerely
